# A Human Serum-Based Enzyme-Free Continuous Glucose Monitoring Technique Using a Needle-Type Bio-Layer Interference Sensor

**DOI:** 10.3390/s16101581

**Published:** 2016-09-24

**Authors:** Dongmin Seo, Sung-Ho Paek, Sangwoo Oh, Sungkyu Seo, Se-Hwan Paek

**Affiliations:** 1 Department of Electronics and Information Engineering, Korea University, Sejong 30019, Korea; ehdals20907@korea.ac.kr (D.S.); swoh@kriso.re.kr (S.O.); 2 Department of Biological Systems Engineering, Virginia Tech, Blacksburg, VA 24061, USA; paek@vt.edu; 3 Maritime Safety Research Division, Korea Research Institute of Ships and Ocean Engineering, Daejeon 34103, Korea; 4 Department of Biotechnology and Bioinformatics, Korea University, Sejong 30019, Korea

**Keywords:** continuous glucose monitoring, competitive assay, glucose dialysis method, bio-layer interferometry, needle-type sensor

## Abstract

The incidence of diabetes is continually increasing, and by 2030, it is expected to have increased by 69% and 20% in underdeveloped and developed countries, respectively. Therefore, glucose sensors are likely to remain in high demand in medical device markets. For the current study, we developed a needle-type bio-layer interference (BLI) sensor that can continuously monitor glucose levels. Using dialysis procedures, we were able to obtain hypoglycemic samples from commercial human serum. These dialysis-derived samples, alongside samples of normal human serum were used to evaluate the utility of the sensor for the detection of the clinical interest range of glucose concentrations (70–200 mg/dL), revealing high system performance for a wide glycemic state range (45–500 mg/dL). Reversibility and reproducibility were also tested over a range of time spans. Combined with existing BLI system technology, this sensor holds great promise for use as a wearable online continuous glucose monitoring system for patients in a hospital setting.

## 1. Introduction

Diabetes mellitus is a metabolic disease in which affected individuals are unable to maintain normal glucose levels [1]. This disorder develops when insulin either cannot be produced by the pancreas or cannot be effectively utilized by cells in the body [2]. In 2013, there were 382 million people suffering from diabetes [3]. By 2030, it is expected that 69% and 20% of all the people in developing and developed countries may be diagnosed as the diabetics [4]. Although diabetes mellitus can be managed with insulin treatment, glucose levels can vary depending on diet and variation in lifestyle. Diabetic patients can monitor glucose levels in their body by carrying out regular blood-glucose tests. A number of blood-glucose monitoring systems are available on the market, such as homecare strip sensors and continuous glucose monitoring sensors. Due to the high demand of such devices, they make up a large portion of the biomedical device market [5]. Strip sensors are commonly used as glucose monitoring devices. Though these sensors have many advantages, including high accuracy and portability, the sample collection procedure (which consists of repeated skin pricking) is very painful [6]. Furthermore, strip sensors provide only a brief snapshot of glucose level at a single moment in time. For the proper management of body glucose levels, tests must be administered for several consecutive days, demonstrating the need for the development of continuous glucose monitoring systems. Continuous glucose monitoring (CGM) systems give a broader view of a patient’s glucose trends by providing continuous data on glucose levels. There are a number of precise and expensive CGM systems available on the market. Most of them are enzyme-based electro-chemical sensors which provide data on electrochemical signals associated with the breakdown and consumption of enzymes [7]. However, since the local glucose concentration can change the permittivity of a medium, the performance of these devices can be influenced by the presence of electromagnetic fields [8].

In this study, we firstly demonstrate the use of a modified competitive assay as an online glucose monitoring system, utilizing the design of needle-type bio-layer interference (BLI) sensor [9]. This enzyme-free optical biosensor measures glucose levels by quantifying wavelength shifts triggered by the binding of glucose to concanavalin A (Con A) molecules. Using commercial human serum, the performance of this system was evaluated for a wide range of glucose concentrations, from hypoglycemia (~45 mg/dL) to hyperglycemia (~500 mg/dL), which fully covers the clinical range of interest (70–200 mg/dL). The results obtained from this platform were compared to the standard BLI system (without needle). The details of the experimental methods and results are discussed in the following sections.

## 2. Materials and Methods

### 2.1. Fabrication of a Novel Needle-Type Sensor

To fabricate the needle-type sensor, a 19-gauge injection needle was cut short to 1.5 cm. The rough surface of the cutting edge was polished using a grinder. The fabricated needle was then cleaned with 70% alcohol and air-dried using a nitrogen gun. The elliptical front opening of the cleaned needle was carefully covered with a polycarbonate semi-permeable membrane (Whatman, Chicago, IL, USA) using a medical-grade epoxy. The hollow space of the needle was then filled with Tris-BSA (Bovine Serum Albumin) buffer. The Con A-immobilized sensor—which was prepared by immobilizing the Con A (Sigma, St. Louis, MO, USA) on the BLI sensor tip via a biotin-streptavidin (Sigma) linkage—was inserted through the open end of the needle. Finally, the space between the needle and sensor surfaces was blocked with silicon glue.

### 2.2. Working Principle of a Newly-Developed Human Serum-Based Needle-Type Sensor

The fabricated needle-type sensor described above quantifies glucose concentration by analyzing the wavelength shift between two reflected lights, one from the protein layer and one from the reference layer. Variation in the thickness of the protein layer results in a wavelength shift in the reflected lights. Because glucose is a small molecule, its binding to Con A limits the signal-to-noise ratio. To address this issue, we used glucose molecules with conjugated ligands (heavy molecule), which competitively bind to the immobilized Con A on the sensor tip. When the needle-type sensor is exposed to a sample containing glucose molecules and conjugates, a concentration gradient of these molecules is produced across the semi-permeable membrane. This force produces a mass transfer of these molecules across the membrane, allowing them to bind competitively to the Con A. The variation of glucose concentration in the sample results in the association or dissociation of conjugates from Con A, which causes the thickness of the bio-layer on the sensor tip to vary. As a result, there is a shift in wavelengths. By studying variation in these associated wavelength shifts, the system can efficiently quantify glucose concentrations in human serum.

### 2.3. Sample Preparation

Commercially available human serum samples contain 100 mg/dL glucose. In order to test sensor performance, we needed serum samples with a wide range of glucose concentrations, representing levels typically associated with both hypoglycemia (<70 mg/dL) and hyperglycemia (>140 mg/dL). We applied the dialysis method to obtain hypoglycemic samples with low glucose levels.

#### 2.3.1. Selective Glucose Dialysis System Used to Obtain Hypoglycemic Samples

The dialysis system used contained a dialysis bag of regenerated cellulose membrane (MWCO 12 kDa, Sigma) and a 50 mL lab beaker with 40 mL of commercial human serum (Sigma). All the sera used in this study were from a same lot (Sigma, Cat. #: H4522-100 mL, Lot #: 8013675226). The dialysis bag was prepared by filling it with a mixture of 40 μL of glucose oxidase (10 μg/mL, Sigma) and 1 mL of human serum. This prepared dialysis bag was clamped and inserted into the beaker containing the human serum. Inside the dialysis bag, glucose molecules react with glucose oxidase (160 kDa) and produce gluconic acid. This process involves the consumption of glucose molecules, which results in the subsequent decrease in glucose concentration inside the dialysis bag, and the formation of a concentration gradient across the dialysis membrane. Due to the formation of this concentration gradient, glucose molecules are diffused from the outside to the inside of the dialysis bag. Hence, the glucose concentration outside the bag decreases. Glucose levels in the serum were checked in regular intervals using a standard strip sensor (FreeStyle, Abbott, Lake Bluff, IL, USA). When the glucose concentration in the serum sample reached 45 mg/dL or 50 mg/dL (hypoglycemia), the dialysis procedure was stopped by removing the dialysis bag.

#### 2.3.2. Preparation of Samples at Various Glucose Concentrations

We prepared four glucose samples with different glucose concentrations (45 mg/dL, 100 mg/dL, 250 mg/dL, and 500 mg/dL) by adding d-glucose (Sigma) to the hypoglycemic serum sample prepared in the section above.

### 2.4. Chemicals, Reagents, and Analytical Components Used

The Octet RED streptavidin (SA) sensor tips were purchased from ForteBio (San Francisco, CA, USA). The polycarbonate membranes of pore sizes 50, 100, and 200 nm were obtained from Whatman (Florham Park, NJ, USA). The 19-gauge syringe needles were supplied by Korea Vaccine Company (Seoul, Korea). Other reagents, including Con A, biotin, d-glucose, bovine serum albumin (BSA), Tris, regenerated cellulose membrane, glucose oxidase, and human serum were obtained from Sigma (St. Louis, MO, USA).

## 3. Results and Discussion

### 3.1. Performance of the Competitive Assay

Using a needle-type sensor, we previously demonstrated the performance of a competitive assay for the measurement of glucose molecules in the presence of mannose-containing glycoproteins (ligand conjugate) [7]. Con A reacts both with glucose and mannose; however, the mannose in plasma is usually stable and negligible in quantity compared to the glucose [10]. It has been reported that fluctuation of glycoprotein level may alter the glucose detection; however, the effect is also relatively minor [11]. In our previous work, the artificial conjugates were placed inside the needle chamber, limiting the pore size of the membrane to 50 nm. This limited pore size influenced the response time of the sensor by inhibiting the permeation rate of the glucose molecules. In addition, we found that the limited migration distance of the ligand conjugate inside the sensor space (inside the needle) affected the overall performance of the system.

**Figure 1:**
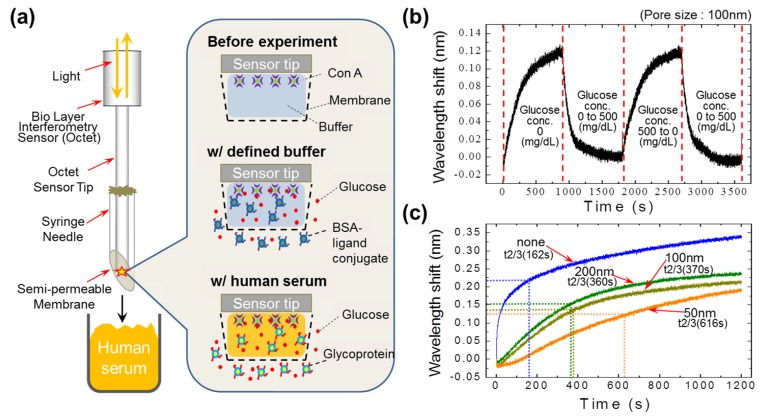
Needle-type sensor performance and the influence of membrane size on the detection of glucose concentrations in defined buffer. (**a**) Schematic of the needle-type sensor. A syringe needle was modified and covered with a semi-permeable membrane to fabricate the needle-type sensor. Bovine serum albumin (BSA)-ligand conjugate was kept outside of the semi-permeable membrane; (**b**) A glucose sensing profile generated by the sensor. The wavelength shift increases with time as the sensor is exposed to the buffer containing the BSA-ligand conjugates, whereas the shift sharply decreases as the sensor is exposed to a solution of 500 mg/dL glucose (with BSA-ligand conjugate); (**c**) Membrane pore-size performance. The response of the sensor increased as the pore size increased from 50 nm to 200 nm. Con A: concanavalin A.

In the present study, we addressed these limitations by placing the ligand conjugate (artificial signal generators) outside of the needle chamber (Figure 1a). This system gave us the freedom to increase the pore size of the membrane to improve the response time of the sensor. Since the ligand conjugates were outside of the sensor space, there was no limitation on migration distance for the conjugate molecules. The performance of the device was studied with a defined buffer of Tris-BSA and ligand conjugate. Following the insertion of the prepared needle sensor into the defined buffer sample, a concentration gradient of ligand conjugate was established across the membrane. Due to this driving force, the conjugates were transferred across the membrane (pore size: 100 nm) and then observed to associate with Con A, resulting in an increased wavelength shift (Figure 1b). When the needle sensor was inserted into the sample containing 500 mg/dL of glucose in the defined buffer, a mass transfer of glucose molecules occurred across the membrane, and then subsequently associated with Con A via the dissociation of the conjugates. This resulted in a decreased shift in wavelength. In human serum, natural conjugates like glycoprotein are available, and are competitively bound to glucose [12]. Based on this, we performed a glucose level detection experiment in human serum, without adding any artificial conjugates.

### 3.2. Performance of the Semi-Permeable Membrane

To optimize pore size for the continuous monitoring of glucose levels, we tested semi-permeable polycarbonate membranes with pore sizes of 50 nm, 100 nm, and 200 nm, and revealed that signal response increases with increasing pore size (Figure 1c). Compared to membranes with small pores (50 nm), those with larger pore sizes better facilitated the mass transfer of molecules; the sensor responses for the 200 nm and 100 nm pore sizes were almost identical. Therefore, to ensure the optimum performance of our system, and to avoid interference from unwanted molecules, we chose the semi-permeable membrane with a pore size of 100 nm.

### 3.3. Production and Screening of Hypoglycemic Samples

**Figure 2:**
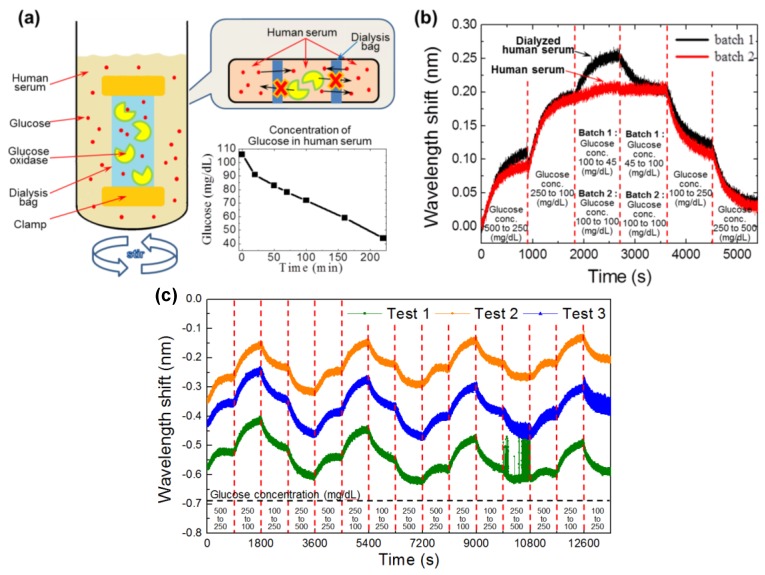
Schematic of the dialysis method and the performance of the sensor for the detection of a broad range of glucose concentrations. (**a**) Schematic of the dialysis procedure. A dialysis bag containing a mixture of glucose oxidase and human serum was used. This dialysis bag was kept in the container of human serum to reduce the glucose concentration of the serum. The time vs. glucose concentration is the glucose attrition of the serum measured with a standard strip sensor; (**b**) Performance of the needle-type sensor. The sensor shows a peak at 1800 s for the batch 1 samples (dialyzed human serum: 45 mL/dL, 100 mL/dL, 250 mL/dL, and 500 mL/dL), which have glucose concentrations comparable to that observed in hypoglycemia, whereas this peak is not observed in the profile for batch 2 (normal human serum: 100 mL/dL, 250 mL/dL, and 500 mL/dL). Averaged standard deviation for a constant glucose concentration (i.e., 100 mg/dL and t = 1800–3600 s of batch 2) was measured as 0.005496 nm, corresponding to 2.77% of the wavelength shift; (**c**) Repeatability test results (*n* = 3) for glucose concentration range of 100–500 mg/dL.

We used the dialysis method to produce a hypoglycemic sample from commercial human serum. The glucose content of the human serum sample was monitored during dialysis using standard strip sensors (Figure 2a). To test the performance of our system for the detection of a range of glucose levels, we prepared two different batches of samples from dialyzed human serum (batch 1: 45 mL/dL, 100 mL/dL, 250 mL/dL, and 500 mL/dL) and normal human serum (batch 2: 100 mL/dL, 250 mL/dL, and 500 mL/dL) by adding d-glucose to obtain the desired concentrations. Performance results for each batch clearly show that our system can sense a broad range of glucose levels (Figure 2b). In Figure 2b, we can see that the 2nd batch samples (which do not have hypoglycemia concentration) show a saturation at 1800 s, whereas the 1st batch samples show a sharp shift in the wavelength at 1800 s due to the presence of the hypoglycemic concentrations (45 mg/dL). In addition, both of the curves showed a decrease in the wavelength shift for the hyperglycemia (500 mg/dL) concentration at 4500 s. The proposed devices (i.e., fabricated needle-type BLI sensors tested in triplicate) showed a fair repeatability for the glucose concentration range of 100–500 mg/dL, as shown in Figure 2c.

### 3.4. Comparison of the Needle-Type Sensor with the Ideal BLI Sensor

**Figure 3:**
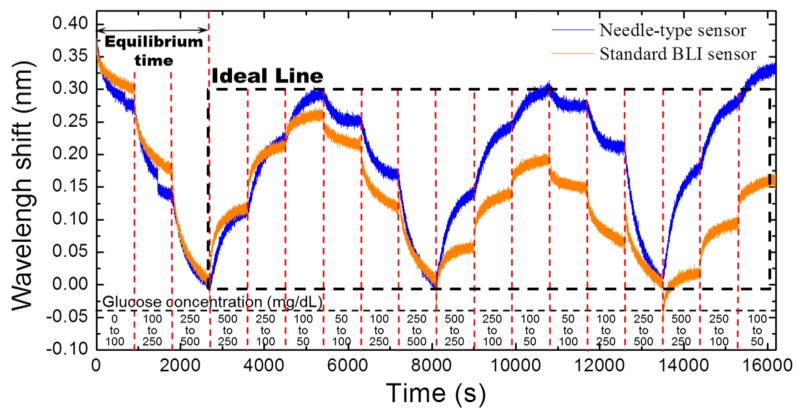
Comparison of the needle-type sensor with the standard bio-layer interference (BLI) sensor. Nearly identical profiles were observed when tested with a wide range of glucose concentrations. The profiles indicate that the reproducibility of the needle-type sensor is more stable than the standard BLI sensor.

We compared the performance of our needle-type sensor with the ideal BLI sensor (Forte Bio, Menlo Park, CA, USA). For that, we used human serum samples with glucose concentrations ranging from 50 mg/dL to 500 mg/dL. The time vs. wavelength shift profiles for these concentrations were obtained using both systems and compared (Figure 3).

We tested the reversibility and reproducibility of the needle-type sensor and compared the results to those obtained by the standard BLI sensor. This analysis revealed that both sensors could detect the varied glucose concentrations after the equilibrium time; however, our system produced more reproducible results. From Figure 3, we can find that the time vs. wavelength shift profile drifted away from the ideal line with time, in contrast to the needle-type sensor profile, which maintained a constant distance from the ideal line throughout the experiment. This difference between the two sensor types can be attributed to interference from unwanted particles. Since the standard BLI sensor tip has an open end, immobilized Con A at the top of the sensor tip is exposed to other particles in the serum. This ultimately affects the overall performance of the sensor after a certain length of time. However, due to the presence of the semi-permeable membrane in the needle-type sensor, immobilized Con A molecules remain protected from unwanted particles. The relatively long period required for the determination of glucose levels—which is due to the equilibrium time (i.e., 900 s in Figure 2 and Figure 3)—may be shortened by employing analytical modeling for those kinetic curves [13].

## 4. Conclusions

In summary, we have firstly demonstrated the utility of a novel needle-type BLI sensor for label-free and continuous glucose monitoring. This sensor was not only able to detect the clinical glucose level of interest (70–200 mg/dL) in human serum, but it could also detect the wider glycemic state range (45–500 mg/dL). Moreover, our device proved to be a stable method for reverse profiling of a wide range of glucose concentrations. The proposed device, as a continuous glucose monitoring tool, has several key advantages: (1) it does not consume any enzymes; (2) it is a label-free optical system; (3) signals generated by the device are free from electromagnetic interference; and (4) artificial conjugates are not required. Thus, we believe that this newly developed needle-type sensor represents an advancement in BMI sensor technology, and can be used as a robust and compact wearable online glucose monitoring system.
